# Upadacitinib’s Effectiveness and Safety as a Second- or Third-Line Therapy in Patients with Ulcerative Colitis: Data from a Real-World Study

**DOI:** 10.3390/jcm14217801

**Published:** 2025-11-03

**Authors:** Giammarco Mocci, Antonio Tursi, Franco Scaldaferri, Daniele Napolitano, Daniela Pugliese, Giovanni Maconi, Giovanni Cataletti, Roberta Pica, Claudio Cassieri, Edoardo Vincenzo Savarino, Caterina De Barba, Francesco Costa, Linda Ceccarelli, Manuela Marzo, Walter Elisei, Rita Monterubbianesi, Roberto Faggiani, Giovanni Lombardi, Marta Patturelli, Davide Giuseppe Ribaldone, Lorenzo Bertani, Stefano Rodinò, Ladislava Sebkova, Giorgia Bodini, Andrea Pasta, Giuseppe Pranzo, Mariaelena Serio, Antonella Scarcelli, Ileana Luppino, Antonio Ferronato, Rocco Spagnuolo, Francesco Luzza, Antonietta Gerarda Gravina, Raffaele Pellegrino, Giuliana Vespere, Silvia Sedda, Vittorio D’Onofrio, Leonardo De Luca, Leonardo Allegretta, Alessia Immacolata Cazzato, Libera Fanigliuolo, Laurino Grossi, Fabio Cortellini, Giacomo Forti, Paolo Tonti, Viviana Neve, Simona Piergallini, Michela Di Fonzo, Federico Iacopini, Pietro Capone, Federica Gaiani, Stefano Kayali, Caterina Mucherino, Elvira D’Antonio, Laura Montesano, Andrea Cocco, Berardino D’Ascoli, Raffaele Colucci, Francesco Bachetti, Giorgia Orrù, Francesca Maria Onidi, Paolo Usai Satta, Marcello Picchio, Alfredo Papa

**Affiliations:** 1 Division of Gastroenterology, AORN “Brotzu” Hospital, 09134 Cagliari, Italy; 2 Territorial Gastroenterology Service, ASL BAT, 76123 Andria, Italy; antotursi@tiscali.it; 3 Department of Medical and Surgical Sciences, School of Medicine, Catholic University, 00168 Rome, Italy; 4 Digestive Diseases Centre (CEMAD), Department of Medical and Surgical Sciences, Istituto di Ricovero e Cura a Carattere Scientifico (IRCCS), Policlinico Universitario “A. Gemelli” Foundation, 00168 Rome, Italy; 5 Gastroenterology Unit, Department Biomedical and Clinical Sciences, “L. Sacco” University Hospital, 20157 Milan, Italy; 6 IBD Unit, Division of Gastroenterology, “S. Pertini” Hospital, 00157 Rome, Italy; 7 Gastroenterology Unit, Azienda Ospedale-Università di Padova (AOUP), 35100 Padua, Italy; 8 IBD Unit, Department of General Surgery and Gastroenterology, Pisa University Hospital, 56124 Pisa, Italy; 9 Division of Gastroenterology, “Veris-Delli Ponti” Hospital, 73020 Scorrano, Italy; manuelamarzo@gmail.com; 10 Division of Gastroenterology, A.O. “S. Camillo-Folanini”, 00151 Rome, Italy; 11 Division of Gastroenterology, AORN “Cardarelli”, 80131 Naples, Italy; 12 IBD Unit, Department of Medical Sciences, Division of Gastroenterology, University of Turin, “A.O.U. Citta della Salute e della Scienza di Torino”, 10126 Turin, Italy; davrib_1998@yahoo.com; 13 Division of Gastroenterology, “Felice Lotti” Hospital, Azienda USL Toscana Nord Ovest, 56025 Pontedera, Italy; lorenzobertani@gmail.com; 14 Division of Gastroenterology, “Ciaccio-Pugliese” Hospital, 88100 Catanzaro, Italy; 15 Department of Internal Medicine and Medical Specialties, Division of Gastroenterology, IRCCS “San Martino” Hospital, University of Genoa, 16132 Genoa, Italy; 16 Ambulatory for IBD Treatment, “Valle D’Itria” Hospital, 74015 Martina Franca, Italy; 17 Division of Gastroenterology, “San Salvatore” Hospital, 61121 Pesaro, Italy; 18 Division of Gastroenterology, “Annunziata” Hospital, 87100 Cosenza, Italy; 19 Digestive Endoscopy Unit, ULSS7 Pedemontana, 36014 Santorso, Italy; 20 Department of Health Science, University of Catanzaro, 88100 Catanzaro, Italy; spagnuolo@unicz.it (R.S.);; 21 Hepatogastroenterology and Digestive Endoscopy Unit, Department of Precision Medicine, University of Campania “Luigi Vanvitelli”, 80138 Naples, Italy; 22 Division of Gastroenterology, “Ospedale del Mare”, ASL Na1, 80147 Naples, Italy; 23 Division of Gastroenterology, “Santa Caterina Novella” Hospital, 73013 Galatina, Italy; 24 Division of Gastroenterology, “S.S. Annunziata” Hospital, 74121 Taranto, Italy; 25 Gastroenterology Unit, “Spirito Santo” Hospital, “G d’Annunzio” University, 65121 Pescara, Italy; 26 Division of Gastroenterology, “Infermi” Hospital, 47921 Rimini, Italy; 27 Division of Digestive Endoscopy, “S. Maria Goretti” Hospital, 04100 Latina, Italy; 28 Division of Gastroenterology, “A. Perrino” Hospital, 72100 Brindisi, Italy; 29 IBD Unit, Division of Gastroenterology, “A. Murri” Hospital, 63900 Fermo, Italy; 30 Division of Gastroenterology, “Ospedale dei Castelli”, 00072 Ariccia, Italy; 31 Division of Gastroenterology, “T. Maresca” Hospital, 80059 Torre del Greco, Italy; 32 Gastroenterology and Endoscopy Unit, Department of Medicine and Surgery, University of Parma, 43126 Parma, Italy; 33 Division of Gastroenterology, Azienda Ospedaliera “S. Anna e S. Sebastiano”, 81100 Caserta, Italy; 34 Division of Gastroenterology, “Madonna Delle Grazie” Hospital, 75100 Matera, Italy; 35 Digestive Endoscopy Unit, “San Matteo Degli Infermi” Hospital, 06049 Spoleto, Italy; 36 Department of Medical Sciences and Public Health, University of Cagliari, 09127 Cagliari, Italy; 37 Division of General Surgery, “P. Colombo” Hospital, ASL Roma 6, 00049 Velletri, Italy; marcellopicchio2@gmail.com

**Keywords:** ulcerative colitis, upadacitinib, real life, remission, safety

## Abstract

**Background:** Upadacitinib (UPA), a selective anti-JAK1 agent, obtained refundability from the Italian National Health System in July 2023 for its use in patients with ulcerative colitis (UC) refractory to other therapies, including anti-TNF-α, anti-integrins, and ustekinumab. At present, no Italian data are available about its effectiveness and safety in the real world. **Methods:** A retrospective assessment of clinical and endoscopic activity was performed according to the Mayo score. The primary endpoints were to evaluate the effectiveness and safety of UPA. **Results:** We included 202 consecutive UC patients (M/F 119/83, median age 42). The clinical remission and clinical response rates were 45.5% (92/202) and 63.5% (128/202), respectively, at 8 weeks and 60.2% and 81.7%, respectively, at the end of the follow-up. Clinical remission was achieved more frequently when UPA was used as a first-line rather than a second-/third-line treatment (*p* = 0.609). Mucosal healing was reported in 84.6% of patients at the median follow-up time. Adverse events occurred in six patients (2.5%), whereas four patients (2%) underwent colectomy. **Conclusions:** This large real-world study shows that UPA is an effective and safe treatment for UC patients.

## 1. Introduction

Inflammatory bowel diseases (IBDs) encompass chronic idiopathic conditions, including ulcerative colitis (UC), Crohn’s disease (CD), and indeterminate colitis (IC). UC, characterized by chronic inflammation of the colonic mucosa, results from a complex interaction between genetic and environmental factors without a clear specific trigger for its occurrence [1]. Since the relapsing and remitting course characterizes UC, sometimes it requires an aggressive therapeutic approach to prevent complications [2].

The advent of biologics, particularly tumor necrosis factor-α (TNF-α) inhibitors, has revolutionized UC management but, unfortunately, up to 30% of patients experience primary non-response and up to 50% experience secondary loss of response or intolerance to these drugs [3,4,5]. Thus, in recent years novel therapeutic agents targeting alternative pathogenetic pathways have been investigated and approved for IBD treatment [6].

Janus kinase (JAK) inhibitors are a family of Tyrosine Kinases (TYKs) that play a pivotal role in cytokine signaling and immune regulation [7]. At present, three JAK inhibitors—filgotinib, tofacitinib, and upadacitinib—have been approved for UC, each of them with different JAK selectivity [8].

Upadacitinib (UPA) is an inhibitor with greater JAK1 selectivity that was granted marketing authorization in 2022 by the European Medicines Agency for the treatment of moderate-to-severe UC in adult patients with inadequate response, loss of response, or intolerance to either conventional therapy or biologics [9].

The efficacy and safety of UPA in UC is supported by pivotal clinical trials, including the U-ACHIEVE and U-ACCOMPLISH trials [10]. Overall, pivotal phase III studies have demonstrated the efficacy and safety of UPA in managing patients with UC, including patients with prior tumor necrosis factor inhibitor failure, and several recent studies have confirmed the drug’s effectiveness in real life [11,12,13,14,15,16,17,18,19,20]. However, these studies were mostly limited by small sample sizes or short follow-ups.

In July 2023, the use of UPA in patients with UC refractory to other therapies, including anti-TNF-α, was also approved in Italy by the Italian Regulatory Agency (AIFA, Agenzia Italiana per il Farmaco) [21].

Thus, the present investigation aimed to assess the effectiveness and safety of UPA in a relatively large cohort of adult patients with UC under real-life conditions and with an adequate follow-up time. We also set out to identify clinical parameters that may influence the response to UPA.

## 2. Materials and Methods

### 2.1. Study Design and Population

We conducted a retrospective, observational, multicenter study on active mild-to-moderate UC outpatients who failed standard or biological therapies (including anti-TNF-a antibodies, vedolizumab, and ustekinumab) or Janus kinase inhibitor (tofacitinib and filgotinib) and were treated with UPA (Rinvoq^®^, Abbvie, Italy) at 37 Italian IBD centers (recognized by The Italian National and Regional Health Systems). All patients who completed at least the induction treatment prior to 31 December 2024 were included.

Eligible patients included men and women at least 18 years of age with an established diagnosis of UC according to standard endoscopic and histological criteria [22]. The exclusion criteria were patients diagnosed with Crohn’s disease or IBD unclassified, those with a prior colectomy, and those in biological combination therapy (i.e., contemporary use of UPA plus another biologic agent or a small-molecule drug).

To collect demographic and clinical data, a shared common database was created. The data collected at baseline were gender, age at diagnosis, smoking habit, extension of the disease, disease duration, previous immunosuppressive and biologic therapies (anti-TNF-α and/or anti-integrin and/or ustekinumab) and/or small molecules (tofacitinib and filgotinib), concomitant medications, fecal calprotectin (FC), C-reactive protein (PCR), erythrocyte sedimentation rate (ESR), total cholesterol, low-density lipoprotein (LDL) and high-density lipoprotein (HDL) cholesterol, triglycerides, creatine kinase (CPK), Mayo score, and Mayo subscore for endoscopy.

The study protocol conformed to the ethical guidelines of the 1975 Declaration of Helsinki as reflected in a priori approval by the institution’s human research committee. All patients gave written informed consent before undergoing endoscopy and UPA treatment. The reference center (Brotzu Hospital, Cagliari, Italy, PROT. PG/2022/18006) obtained the ethics committee approval for this retrospective study and was accepted by the other centers.

### 2.2. Study Treatment

UPA (RinvoqTM) is approved in Italy for patients with UC only after the failure of first-line biotechnological treatment, except for patients with contra-indications to other biotechnological agents [21]. All patients were treated uniformly during the induction phase with 45 mg once daily for eight weeks via the oral route to obtain remission. In case of a lack of clinical remission at week 8, an additional treatment period of 8 weeks with UPA 45 mg once daily was allowed according to the prescription’s rules.

After induction, a 30 mg or 15 mg once-daily dosage was used to maintain remission in case of clinical response. The highest dose for maintenance treatment was chosen by all the investigators, considering that most of the patients had experienced failure of one or more biological agents.

Finally, treatment discontinuation and the chance to use an 8-week course again at 45 mg once daily during the follow-up to re-induce remission were considered. Also, concomitant medications, such as oral and topical aminosalicylates, steroids, and/or immunosuppressants, were left to the investigators’ judgment.

### 2.3. Clinical Assessment at Baseline and During the Follow-Up

The Montreal classification [22] was used to assess the disease extension while the Mayo score [22] was used to determine the severity of the disease. Active disease despite concomitant treatment was defined as a Mayo score ≥ 3 points [23]. Patients were clinically evaluated at entry and then after two, four, six, nine, and twelve months. CRP and FC levels were obtained at baseline and then at two, four, six, nine, and twelve months, or in case of a loss of clinical response. Finally, based on findings that JAKs can alter the lipid profile and CPK levels [24], total cholesterol, LDL, HDL, triglycerides, and CPK were evaluated at baseline, within two months of starting, and, in case of altered values, during the follow-up.

In addition, the number of days required (during the first eight weeks of treatment) to obtain the disappearance of rectal bleeding, abdominal pain, and urgency was assessed using daily patient-reported outcomes (PROs).

### 2.4. Endoscopy Assessment at Baseline and During the Follow-Up

As an inclusion criterion for this study, all enrolled patients underwent an ileo-colonoscopy before starting UPA treatment. During the follow-up, after a minimum of 6 months since enrollment in the study, patients underwent ileo-colonoscopy according to the clinician’s judgment. The Mayo subscore for endoscopy was used to assess the endoscopic severity [23]. No central reading for assessment of the endoscopic activity was available.

### 2.5. Outcomes

The primary outcome assessed in this study was the effectiveness of UPA for the treatment of patients with UC refractory to standard therapies, anti-TNFα and/or anti-integrin drugs and/or IL 12/23 and/or small molecules, or steroid-dependent in terms of clinical remission, defined as a partial Mayo score (PMS) ≤ 1 (with no single item scoring > 1) [25], at eight weeks and the end of the follow-up. We defined UPA use as first-, second-, or third-line when used for those naïve to biologics (first-line therapy) and resistant or refractory to steroids, or when used in patients who failed one or more anti-TNF-α treatments (second-line therapy), or when used in patients who failed anti-TNF-α and anti-integrin or anti-IL12/23 treatment (third-line therapy).

The co-primary outcome was the safety of UPA, assessed by recording adverse events (AEs). The AEs were classified as early (occurring during the first eight weeks of treatment) or late (occurring after this date) and graded as mild (not requiring the therapy to stop) or severe (requiring a stop to treatment). The occurrence of opportunistic infection was also regarded as an AE. This was defined as an infection caused by microorganisms with limited pathogenic capacity under normal circumstances. Such infections can cause disease because of the predisposing effect of another disease or its treatment [26].

In addition, this study had several secondary outcomes:

- Clinical response at week eight and at the end of follow-up, defined as a decrease of at least two points in the PMS compared to baseline [25];
- Steroid-free clinical remission at week 8 and at the end of follow-up, defined as a PMS ≤ 1 and the absence of steroid treatment [25];
- Bowel urgency response, defined as the number of days from the start of UPA to a reduction in or the disappearance of bowel urgency;
- Change in steroid use (defined as the rate of steroid use over the total number of patients in the time window considered) during follow-up;
- UPA optimization, defined as an extension of induction to 16 weeks with UPA 45 mg once daily in case of a lack of clinical remission at week 8, or the chance to use an 8-week course again at 45 mg once daily during the follow-up to re-induce remission;
- Mucosal healing (MH) at the end of follow-up (defined as a Mayo endoscopic subscore ≤ 1) [25];
- Rate of colectomy during follow-up;
- CRP, FC, and PMS variations during follow-up;
- CRP, FC, and PMS short-term variations between 0 and 8 weeks according to the number of previous biological therapies performed;
- Clinical response during follow-up.

### 2.6. Statistical Analysis

MedCalc Release 14.8.1 was used to analyze the data. The study group’s characteristics were examined as the median (interquartile range [IQR]) for continuous non-parametric variables and as a number (percentage) for categorical variables. The Chi-square test was used to compare categorical variables, and the Mann–Whitney test was used for continuous variables. Clinical remission was considered the primary endpoint. Because of the varying length of follow-up, the predictive value of clinical parameters was assessed using time-to-event methods for censored observations. The duration of follow-up was calculated from the date of starting therapy to the date of the event or censorship. Time-to-event analysis was carried out using Kaplan–Meier estimates to draw the cumulative incidence curves, compared by log-rank tests. Prognostic variables were analyzed by univariate and multivariate Cox’s proportional hazards (PH). *p*-values of <0.05 were considered to be statistically significant.

## 3. Results

### 3.1. Baseline Characteristics

The study group comprised 202 patients (58.9% males, with a median [IQR] age of 42.7 [29.8–51.7] years). The median follow-up length was 4 (IQR 3–6) months. The median Mayo score and median MES were 6 and 3, respectively. The characteristics of the study group are reported in Table 1. Most patients suffered from pancolitis (59.4%) and were treated with UPA after the failure of anti-TNFα plus anti-integrin or anti-IL12/23 (63.4%). Finally, 53 patients (26.3%) were previously exposed to another Jak inhibitor.

### 3.2. Primary Outcomes

**Figure 1:**
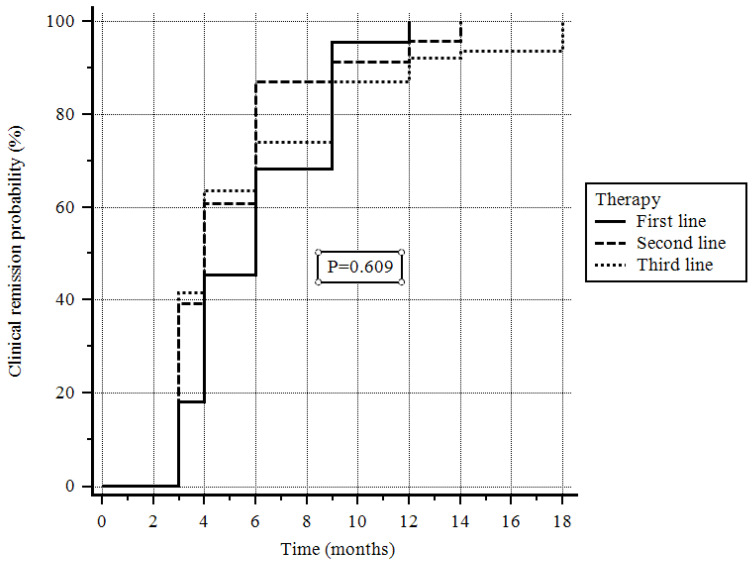
Probability of clinical remission depending on the line of treatment.

At the 8-week follow-up, clinical remission was achieved in 92 (45.5%) patients; at the end of follow-up, clinical remission was achieved in 122 (60.4%) patients. In particular, clinical remission was obtained by 22/31 (71.0%) of patients on first-line therapy, by 23/43 (53.58%) of patients on second-line therapy, and by 77/128 (60.2%) of patients on third-line therapy (*p* = 0.609, log-rank test; Figure 1).

The clinical remission rates in the cohort that had failed a first JAKi, mainly tofacitinib, were 47% (25/53) at week 8 and 56.6% (30/53) at the maximum follow-up.

In univariate analysis, clinical remission at the 8-week follow-up (HR = 2.91 [95%CI = 2.04–4.17], *p* = 0.000) significantly predicted final clinical remission, while a PMS > 6 (HR = 0.54 [95%CI = 0.38–0.77], *p* = 0.000) and a Mayo subscore for endoscopy > 2 (HR = 0.71 [95%CI = 0.50–1.02], *p* = 0.026) were negative factors for clinical remission. In multivariate analysis, clinical remission at the 8-week follow-up (HR = 2.95 [95%CI = 1.96–4.47], *p* = 0.000) was the only factor predicting clinical remission, while a PMS > 6 (HR = 0.57 [95% CI = 0.38–0.87], *p* = 0.008) was the single negative predictive factor for clinical remission (Table 2).

### 3.3. Secondary Outcomes

Clinical response at week eight and at the end of follow-up was achieved in 128 (63.5%) and 165 (81.7%) patients, respectively. On endoscopic assessment, mucosal healing was observed in 44/52 (84.6%) patients. Steroid-free remission was present in 83/202 (41%) at week 8 and 113/122 (92.6%) patients at the end of follow-up. Surgery was necessary in four (2.0%) patients.

After the initiation of UPA, the disappearance of UC-related symptoms was achieved as follows: diarrhea in 10 (IQR 5–15) days, abdominal pain in 7 (IQR 4–13) days, and urgency in 10 (IQR 5–15) days.

During the follow-up, 157 (77.7%) patients were steroid-free, and UPA optimization was performed in 82 (40.6%) patients.

**Figure 2:**
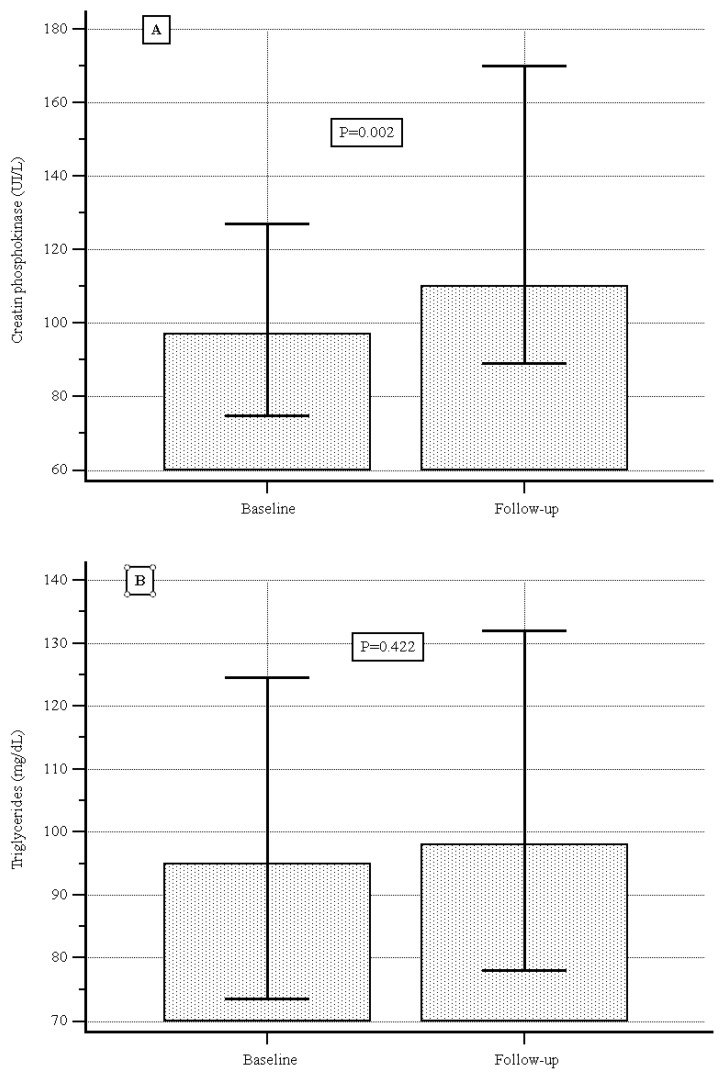

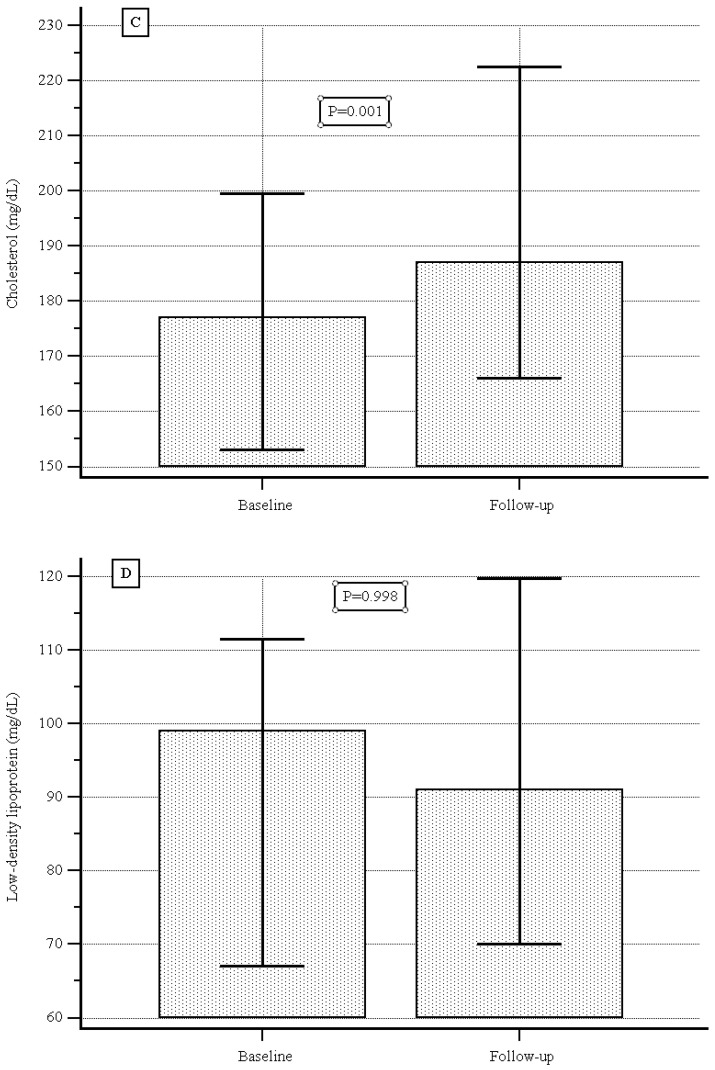
(**A**) Creatine phosphokinase levels at baseline and during follow-up. (**B**) Triglyceride levels at baseline and during follow-up. (**C**) Cholesterol levels at baseline and during follow-up. (**D**) Low-density lipoprotein at baseline and during follow-up.

At baseline the median (IQR) serum CPK level was 97.0 (89.2–101.0) UI/L, and during the follow-up it was 110.0 (98,0–130) UI/L, showing a significant increase (*p* = 0.02, Figure 2A); the median (IQR) serum cholesterol level was 177.0 (169.3–186.0) mg/dL vs. 187.0 (180.0–200.0) mg/dL, showing a significant increase (*p* = 0.001, Figure 2B); no significant difference was detected with respect to serum levels of triglycerides or low-density lipoproteins (Figure 2C,D).

**Figure 3:**
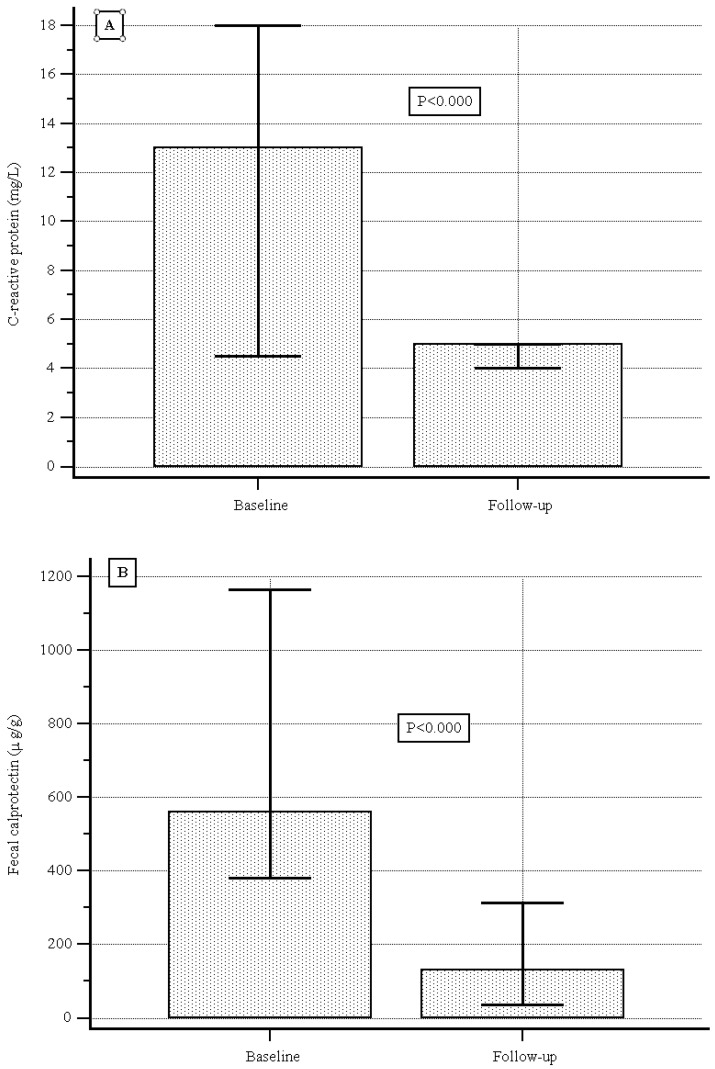
(**A**) C-reactive protein levels at baseline and during follow-up. (**B**) Fecal calprotectin levels at baseline and during follow-up.

At baseline the median (IQR) serum C-reactive protein level was 13.0 (4.5–18.0) mg/L, and during follow-up it was 5.0 (4.0–5.0) mg/L, showing a significant decrease (*p* < 0.000, Figure 3A); the median (IQR) fecal calprotectin level was 560.0 (380.0–1166.0) µg/g vs. 130.0 (34.5–312.0) µg/g, showing a significant decrease as well (*p* < 0.000, Figure 3B).

**Figure 4:**
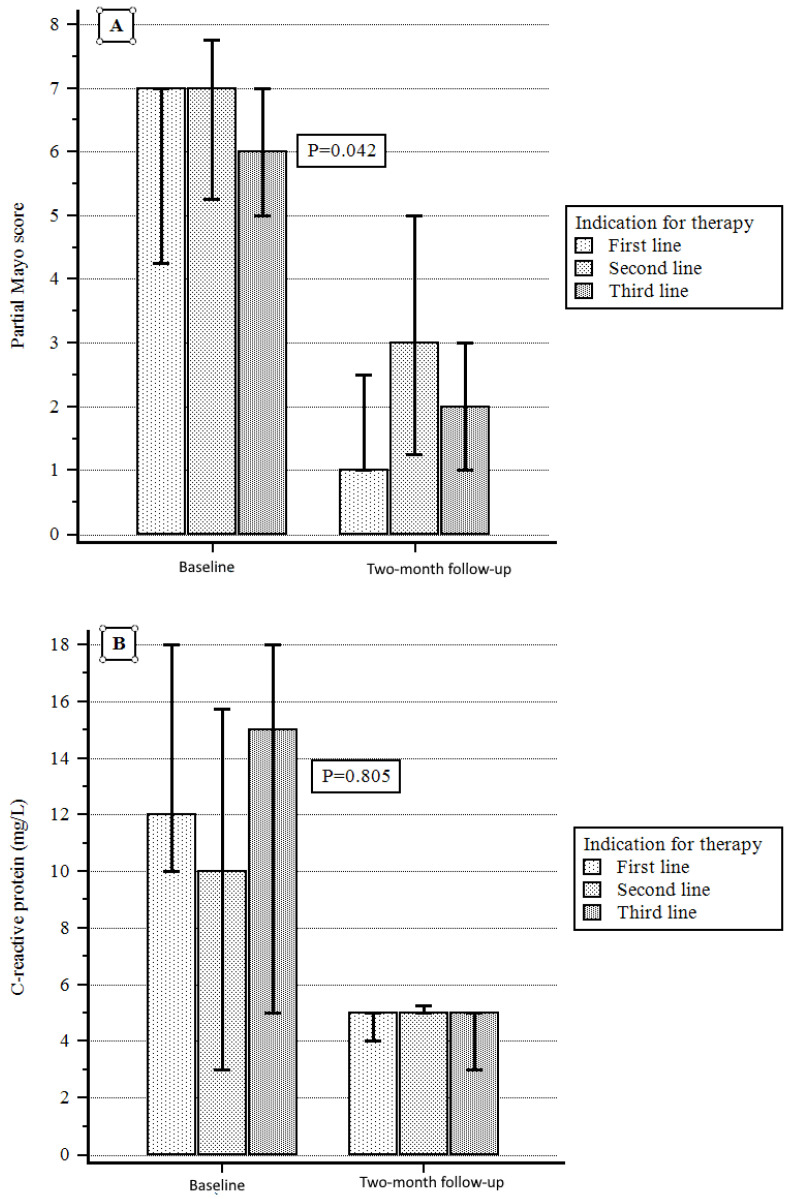

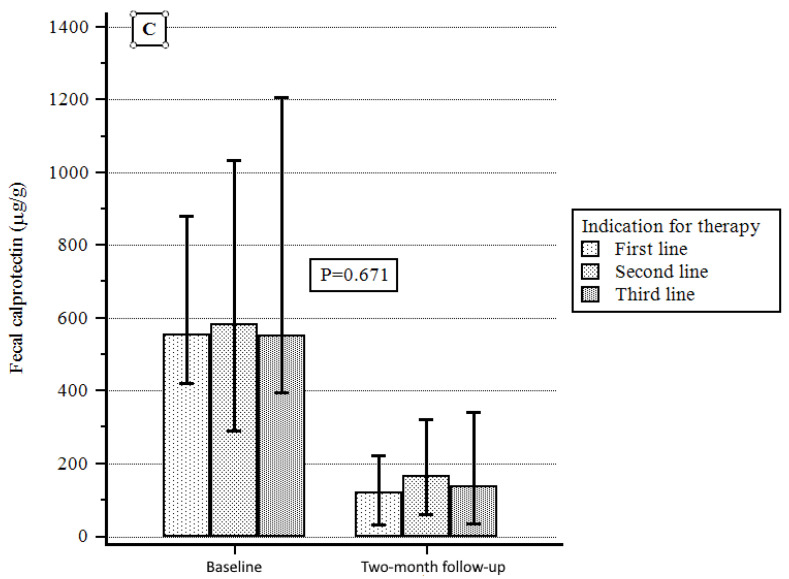
(**A**) PMS variation according to indication for therapy. (**B**) C-reactive protein variation according to indication for therapy. (**C**) Fecal calprotectin variation according to indication for therapy.

According to the indications for therapy, a significant reduction in PMS was obtained in the first-line therapy group with respect to the others from baseline to the two-month follow-up, while a non-significant difference was observed when considering either C-reactive protein or fecal calprotectin variation (Figure 4A–C).

### 3.4. Adverse Events

Table 3 reports AEs. The adverse event rate was very low, and such events were recorded in only six patients (2.5% of the population): in four cases (1.98% of the population) they were mild and did not require the suspension of UPA treatment; in two cases (0.49%) they were severe and required a stop to UPA administration (see Table 3). Significantly, no instances of Major Adverse Cardiovascular Events (MACEs) or venous thromboembolic events (VTEs) were recorded. Of the two severe cases, one patient had pneumonia, and the other patient was admitted to the Emergency Room with a fever of >39 °C and therefore admitted to the Division of Infectious Diseases with a diagnosis of “Generalized Infection”. The UPA treatment was stopped, but no further information was drawn because the patient was lost to follow-up after discharge. The two severe AEs occurred after 3 and 5 months of treatment, respectively. In both cases, the patient did not have cardiovascular disease at baseline.

## 4. Discussion

Even though over the past decade the therapeutic landscape has rapidly evolved, the current management of UC remains challenging due to low response rates, primary failure, loss of response, and intolerance. JAK inhibitors are a novel group of small-molecule inhibitors that have demonstrated efficacy in inducing and maintaining remission in moderate-to-severe UC [26]. In particular, UPA, an inhibitor with greater JAK1 selectivity approved in 2022 [9], has demonstrated efficacy in inducing and maintaining remission in UC in pivotal studies and, as comparative efficacy trials are lacking, has been shown to be superior to other therapies in achieving clinical remission, endoscopic improvement and remission, and histological remission [27,28]. Furthermore, several real-world studies (RWSs) have confirmed the drug’s effectiveness and safety in real life; however, these investigations were limited by several drawbacks, mainly small sample sizes and short follow-ups [11,12,13,14,15,16,17,18,19,20,21].

In this real-life study, we enrolled a consistent number of patients, considering the relatively short time since UPA approval for UC in Italy [22]. At baseline, most patients had received previous biologic treatments, and more than half had experienced the failure of drugs with two or more mechanisms of action. Furthermore, about a quarter of the patients had already been treated with another Jak inhibitor.

First, our real-world analysis supports the effectiveness of UPA in a real-life scenario in achieving clinical, biochemical, and endoscopic remission. The primary endpoint, clinical remission at week 8, was achieved in 45.5% of the patients, a better rate than those reported in the pivotal studies ACHIEVE induction [UC1] and U-ACCOMPLISH [UC2] [10], in which patients achieving clinical remission accounted for 26% and 34%, respectively. As drugs often perform better in real life than in trials, it is interesting to observe how, in our study, the median disease duration was higher than those in pivotal studies and, especially, how only 15% of the patients were bio-naïve, compared to about 50% in UC1 and UC2.

Moreover, our data are only partially in line with the other real-world studies (RWSs) published worldwide. For instance, in a multicenter Australian cohort of 152 patients with moderate-to-severe UC, the primary endpoint of clinical remission was met in 79% at week 8 and 84% at week 16 [29]. In a recent meta-analysis of twenty-four studies with 1388 patients included, of which 3 out of 4 had previously failed two or more biologics, inductions of clinical response and remission were achievable with UPA in 82% and 65% of patients, respectively [30]. At least in part, these findings could be explained by the strict criteria for defining remission adopted for our study. Indeed, we defined remission as PMS ≤1: this means that patients must be almost asymptomatic (or asymptomatic) to be considered “in remission”. This condition is difficult to achieve in real life, where patients are usually more complex, for example, due to the presence of comorbidities and the use of medications that can affect bowel habits. In this regard, it is significant that over 90% of patients in clinical remission by week 8 had also achieved steroid-free remission. However, we should also consider that the results of this meta-analysis suffer from several biases, ranging from high heterogeneity (>80%) in achieving remission at week eight to limited sample sizes in some included studies.

Moreover, subgroup analysis evaluated the effectiveness of UPA as a second JAKi for UC. Showing similarities with a recent real-life study [31,32], in our results, outcomes with UPA were not compromised by prior JAKi failure, with nearly 47% of such patients achieving the primary endpoint. In particular, Akiyama et al. [32], given the higher efficacy of upadacitinib even in patients previously exposed to biologics or other JAK inhibitors, suggested that this drug can be used as a second- or third-line JAK inhibitor.

Second, in our cohort the remission rate increased during treatment with UPA, rising from 45.5% at 8 weeks to 60.4% at the end of the follow-up. This time-dependent effectiveness trend not only confirms the real-life experiences published so far on UPA [30] but also is similar to what our group has observed for the other two JAKs, tofacitinib and filgotinib [33,34]. Alongside the speed of action of the drug, we believe that this is a crucial point: it is important to continue the treatment with UPA until the 24th week, since the remission rate increases progressively. To reinforce this concept, during follow-up there was a reduction in steroid use, with more than 92% of patients in clinical remission also being steroid-free.

Another important point arising from this study is that, though in a statistically insignificant manner, the clinical remission rate differed according to the setting of UPA. In fact, we observed remission rates of 71% in patients naïve to any biotechnological therapy, 53% in patients receiving second-line therapy, and 60% in patients receiving third-line therapy. Again, in the Italian population this behavior appears to be the same as that of other Jak inhibitors [33,34], which work better in bio-naïve than in bio-experienced UC patients. Moreover, this result seems to be different to that reported in a recent study that reported JAK inhibitors to be more effective in anti-TNF-exposed than in anti-TNF-naïve patients, based on six RCTs [35]. There are several possible interpretations for this difference. First of all, it is always difficult to compare the results of large RCTs with those from real life: there are too many differences in the inclusion and exclusion criteria, comorbidities, and scores used to assess whether there is a response to a particular treatment. Another potential hypothesis might relate to the intrinsic characteristics of the population (e.g., diet or genetics) enrolled. Furthermore, another possible explanation is that a high expectation towards the therapy could justify better effectiveness in naïve patients (placebo effect), while patients who have already had a biotechnological therapy fail could have a more negative approach to a new therapy (nocebo effect). Whatever the explanation may be, in our population UPA worked better in patients naïve to biotechnological therapies.

We also analyzed factors that might predict final clinical remission with UPA. We found that the presence of remission at the 8-week follow-up was the only predictor of remission in a multivariate analysis, whereas a partial Mayo score > 6 was the single negative predictive factor for clinical remission.

Looking at the secondary endpoints, among serum lipid levels, at baseline the total cholesterol, LDL cholesterol, and triglyceride levels were within average values. During follow-up we recorded a slight increase in total cholesterol but not in triglycerides or LDL cholesterol.

One of the strengths of Jak inhibitors is definitely their speed of action. Our results confirmed the fast onset of action with upadacitinib [36], with the disappearance of bowel urgency and diarrhea 10 days after the initiation of UPA. Moreover, the effectiveness of UPA is supported by the normalization of objective biomarkers of efficacy. Both CRP and CF significantly dropped during the follow-up, and MH was present in almost 85% of patients—a very positive result that, however, must be interpreted with caution since only a quarter of our population underwent endoscopy. Finally, 82 (40.6%) patients required dose optimization. This finding is relevant to practice and should encourage clinicians to continue induction therapy with 45 mg for 16 weeks, especially in more difficult patients, such as those who have previously failed other biological therapies or have more severe disease activity. On the other hand, after this timepoint, in the absence of clinical signs of efficacy and with objective markers of disease activity, another therapeutic option should be considered.

The safety profile of UPA was good and consistent with the established safety profile from pivotal RCTs and long-term extension studies in UC [10,37]. During follow-up, AEs occurred in only five (2.5%) patients, and in only one case, the AEs were severe and required treatment withdrawal. Significantly, although the follow-up period was relatively short, no instances of venous thromboembolism, MACEs, or malignancies were recorded. No case of simplex herpes zoster was recorded. Finally, overall, our safety data are better than those in the current literature arising from the real world, which report incidence rates of serious adverse events and herpes zoster of 2.3 and 1.7 per 100 patient-years, respectively [29]. We cannot exclude that the retrospective design of this study might have missed some mild AEs. On the other hand, our results align with several experiences showing a low prevalence of serious AEs, thus confirming the excellent safety profile of UPA.

Our study has several strengths. The main strength lies in the large number of patients enrolled in this real-life study and the use of standardized clinical scores to evaluate disease activity. Strict criteria defining remission and clinical response enhanced the results obtained. In addition to its strengths, the limitations of this study must be acknowledged. First, its retrospective design inherently carries a risk of missing data, especially at later follow-up timepoints. Second, the short-term follow-up (24 weeks) may represent a limitation. Third, as already mentioned, another limitation due to the retrospective design was that some mild AEs could have been missed; this risk, however, does not exist for severe events, which are more easily recognized and reported. Finally, endoscopic assessments were unavailable for a substantial proportion of patients, limiting conclusions regarding mucosal healing.

## 5. Conclusions

In conclusion, this real-life study found that UPA can be used to effectively and safely manage outpatients with UC. The clinical effectiveness significantly differs according to the setting of UPA use, with a particular benefit in bio-naïve patients, contrary to what was previously reported in the literature. Interestingly, we identified some parameters that can help physicians predict UPA’s effectiveness.

Obviously, further studies with large sample sizes, a longer follow-up, and prospective designs are needed to confirm these findings.

## Figures and Tables

**Table 1:** Characteristics of the study group (202 patients).

Male sex	119 (58.9)
Median (IQR) age at diagnosis, years	42.7 (29.8–51.7)
Median (IQR) disease duration prior to upadacinitib use, years	8 (4 to 15)
Current smokers	33 (16.3)
Presence of comorbidities	96 (47.5)
Appendectomy	7 (3.5)
Previous surgery	8 (4.0)
Median (IQR) Mayo score	6 (5–7)
Median (IQR) Mayo endoscopic score	3 (2–3)
Extent of disease:	
Proctitis (E1)	7 (3.5)
Left-sided colitis (distal colitis included, E2)	75 (37.1)
Pancolitis (E3)	120 (59.4)
**Previous conventional therapy**	
Mesalazine	200 (99.2)
Steroids	192 (95.1)
Tiopurine	63 (31.2)
**Indications for therapy with upadacinitib**:	
Steroid-dependent/-resistant (first line)	31 (15.3)
Failure of anti-TNFα treatment (second line)	43 (21.3)
Failure of anti-TNFα plus anti-integrin or anti-IL12/23 (third line)	128 (63.4)
**Prior advanced drug therapy**	
Infliximab	161 (79.7%)
Adalimumab/Golimumab	60 (29.7%)/18 (8.9%)
Vedolizumab	82 (40.6%)
Ustekinumab	46 (22.8%)
Tofacitinib	47 (23.3%)
Filgotinib	6 (3%)
**Concomitant therapy**	
Mesalazine	197 (97.5)
Steroids	40 (19.8)
Thiopurine	2 (1.1)
Median (IQR) creatine phosphokinase level (UI/L)	97.0 (74.7–127.0)
Median (IQR) cholesterol level (mg/dL)	177.0 (153.0–199.5)
Median (IQR) LDL level (mg/dL)	99.0 (67.0–111.5)
Median (IQR) triglyceride level (mg/dL)	95.0 (73.5–124.5)
Median (IQR) CRP (mg/L)	13.0 (4.5–18.0)
Median (IQR) calprotectin (µg/g)	560 (380.0–1166.0)

Data are given as number (percentage) of patients unless otherwise indicated. IQR, interquartile range.

**Table 2:** Association of clinical characteristics with remission.

	Remission		Univariate Analysis		Multivariate Analysis	
Variables	Yes	No	HR (95% CI)	*p*	HR (95% CI)	*p*
Sex						
Male	71 (59.7)	48 (40.3)	Ref.		.	
Female	51 (61.5)	32 (38.5)	0.97 (0.68–1.39)	0.842	0.98 (0.68–1.42)	0.921
Age						
≤40 yrs	55 (60.9)	36 (39.1)	Ref.		-	
>40 yrs	66 (60.0)	44 (40.0)	1.02 (0.72–1.46)	0.881	1.04 (0.69–1.57)	0.843
Disease duration						
≤8 yrs	62 (57.4)	46 (42.6)	Ref.			
>8 yrs	60 (63.8)	34 (36.2)	0.98 (0.69–1.40)	0.901	0.89 (0.59–1.35)	0.578
Smoking						
No	101 (59.8)	68 (40.2)	Ref.		-	
Yes	21 (63.6)	12 (36.4)	0.86(0.53–1.42)	0.476	1.16 (0.71–1.90)	0.540
Comorbidities						
No	66 (62.3)	40 (37.7)	Ref.		-	
Yes	56 (58.3)	40 (41.7)	1.22 (0.86–1.74)	0.185	0.84 (0.58–1.22)	0.358
Extent of the disease						
Proctitis/left-side colitis (E1/E2)	46 (56.1)	36 (43.9)	Ref.			
Pancolitis (E3)	76 (63.3)	44 (36.7)	1.03 (0.71–1.48)	0.863	0.96 (0.64–1.42)	0.837
Line of treatment						
First line	45 (60.8)	29 (38.1)	Ref.		-	
Second/Third line	77 (60.2)	51 (39.8)	1.06 (0.73–1.54)	0.707	0.98 (0.64–1.50)	0.926
Partial Mayo score (PMS)						
≤6	79 (71.8)	31 (28.2)	Ref.		-	
>6	43 (46.7)	49 (53.3)	0.54 (0.38–0.77)	0.000	0.57 (0.38–0.87)	0.008
Mayo endoscopic subscore						
≤2	68 (68.7)	31 (31.3)	Ref.		-	
>2	54 (52.4)	49 (47.2)	0.71 (0.50–1.02)	0.026	0.88 (0.60 to 1.32)	0.558
Clinical remission at 2 months						
No	38 (34.5)	72 (65.5)	Ref.		-	
Yes	84 (91.3)	8 (8.7)	2.91 (2.04–4.17)	0.000	2.95 (1.96–4.47)	0.000

Data are given as number (percentage) of patients. HR, Hazard Ratio. *p*, log-rank test.

**Table 3:** Rate and type of adverse events recorded.

	Total N (%)
**Total AEs**	6 (2.5)
**Mild–Moderate AE**	4 (80)
- Cough	1
- Salmonella P. infection	1
- Hypertransaminasemia	1
- CPK elevation	1
**Severe AE**	2 (20)
- Pneumonia	1
- Infection	1

## Data Availability

Data from this study are available from the corresponding author upon reasonable request.
